# Sarcoplasmic Hypertrophy in Skeletal Muscle: A Scientific “Unicorn” or Resistance Training Adaptation?

**DOI:** 10.3389/fphys.2020.00816

**Published:** 2020-07-14

**Authors:** Michael D. Roberts, Cody T. Haun, Christopher G. Vann, Shelby C. Osburn, Kaelin C. Young

**Affiliations:** ^1^School of Kinesiology, Auburn, AL, United States; ^2^Department of Cell Biology and Physiology, Edward Via College of Osteopathic Medicine – Auburn Campus, Auburn, AL, United States; ^3^Fitomics, LLC, Birmingham, AL, United States

**Keywords:** muscle hypertrophy, resistance training, sarcoplasm, myofibril, myofiber

## Abstract

Skeletal muscle fibers are multinucleated cells that contain mostly myofibrils suspended in an aqueous media termed the sarcoplasm. Select evidence suggests sarcoplasmic hypertrophy, or a disproportionate expansion of the sarcoplasm relative to myofibril protein accretion, coincides with muscle fiber or tissue growth during resistance training. There is also evidence to support other modes of hypertrophy occur during periods of resistance training including a proportional accretion of myofibril protein with fiber or tissue growth (i.e., conventional hypertrophy), or myofibril protein accretion preceding fiber or tissue growth (i.e., myofibril packing). In this review, we discuss methods that have been used to investigate these modes of hypertrophy. Particular attention is given to sarcoplasmic hypertrophy throughout. Thus, descriptions depicting this process as well as the broader implications of this phenomenon will be posited. Finally, we propose future human and rodent research that can further our understanding in this area of muscle physiology.

## Introduction

Research ranging from the latter part of the 20th century to today has shown that resistance training increases radial (or cross-sectional) skeletal muscle fiber growth (reviewed in Folland and Williams, 2007; Walker et al., 2011; Haun et al., 2019c). However, less is known regarding the morphological adaptations that occur in muscle fibers following resistance training interventions. Our group recently posited that higher volume resistance training may facilitate fiber growth through a disproportionate increase in the volume of sarcoplasm relative to myofibril protein accretion (Haun et al., 2019b; Vann et al., 2020b). *Sarcoplasmic hypertrophy* has been a mainstream description of this phenomenon, and this term will be used herein. We are not the first to suggest that sarcoplasmic hypertrophy occurs in response to resistance training, and studies cited later in this review have supported this mechanism. However, it is generally accepted by the scientific community that *conventional hypertrophy* occurs in response to resistance training; specifically, muscle fibers undergo training-induced radial growth through a proportional accretion of myofibrillar protein and sarcoplasm space. Under this pretense, if an individual exhibits a 20% increase in mean fiber cross-sectional area (fCSA), and assuming myofibrils constitute ∼85% of intracellular space, a 17% addition of myofibrillar protein and a 3% increase in sarcoplasm volume would accompany fiber growth. This mode of muscle fiber hypertrophy is certainly sensible. Notwithstanding, there is only one human study to date which loosely supports this mechanism (Luthi et al., 1986), and this study is discussed in greater detail later in the review.

Numerous studies have reported myofibrillar protein synthetic rates increase hours following a training bout in untrained (reviewed in Walker et al., 2011; Haun et al., 2019c) and trained persons (reviewed in Damas et al., 2015). These observations have been consistent and certainly lend credence to the conventional hypertrophy model discussed above. While informative, tracer studies have not resolved the degree to which long-term myofibril protein accretion contributes to muscle fiber hypertrophy. In fact, post-exercise increases in myofibrillar protein synthesis rates hours following an exercise bout are unrelated to long-term hypertrophic outcomes (reviewed in Mitchell et al., 2015). Studies examining integrated myofibrillar protein synthesis rates over days or weeks into training have yielded better associations with hypertrophic outcomes (Brook et al., 2015; Damas et al., 2016a; Bell et al., 2019). Nonetheless, it remains possible that the variance existing between these tracer data and hypertrophic outcomes could be related to the growth of non-myofibril components. Moreover, no human investigation has determined whether training-induced myofibril protein accretion occurs via the enlargement of pre-existing myofibrils, the creation of new myofibrils (i.e., *de novo* myofibrillogenesis), or the enlargement of myofibrils followed by myofibril splitting to maintain a conserved myofibril size. These are knowledge gaps in the resistance training literature to be certain. Rather than focusing the current review on these drawbacks, however, we aim here to provide a detailed and in-depth review of scenarios where sarcoplasmic hypertrophy has and has not been observed in the human resistance training literature. We also aim to explain why this phenomenon may occur from a theoretical and mechanistic perspective. Finally, assuming sarcoplasmic hypertrophy is a resistance training adaptation, we discuss the broader implications of this mechanism.

## Intracellular Morphology of Muscle Fibers

**FIGURE 1:**
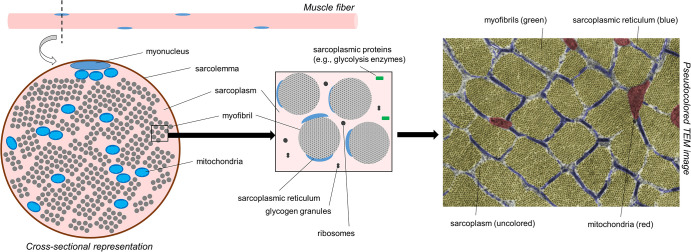
A simplified schematic of muscle fiber morphology. The left inset is a recreated figure of what would be observed under a TEM if tissue were prepared for cross-sectional imaging. The right inset image is a pseudocolored cross-sectional TEM image within the skeletal muscle fiber of a Wistar rat showing different cellular components (15,000×). Notably, much of the attention in this figure is devoted to myofibrils. However, it is also important to note that mitochondria make up ∼5% of the muscle cell area in cross section. The image was licensed from sciencesource.com (courtesy of Jose Luis Calvo) by the authors for scientific publication purposes.

Prior to discussing the different modes of hypertrophy, it is critical for readers to appreciate the intracellular morphology of muscle fibers. Figure 1 illustrates the cross-section of a muscle fiber as it would appear under a transmission electron microscope (TEM). From a molecular perspective, our laboratory has recently reported that muscle tissue is made up of ∼75% water, ∼10–15% myofibrillar protein, and ∼5% non-myofibrillar (or sarcoplasmic) protein (Vann et al., 2020b). From a spatial perspective, however, a majority of the muscle fiber is occupied by myofibrils; some estimates suggest myofibrils occupy ∼85% of the intracellular space (Macdougall et al., 1982; Alway et al., 1988; Claassen et al., 1989). Myofibrils consist of various proteins including myosin heavy chain and light chain isoforms, various actin isoforms, various troponin and tropomyosin isoforms, z-line proteins (e.g., alpha-actinin, muscle-specific LIM protein, myopalladin, and telethonin amongst others), m-line proteins (e.g., myomesin and M-protein amongst others), and other proteins which serve to maintain the structural integrity of myofibrils (e.g., titin and nebulin amongst others) (Haun et al., 2019c; Vann et al., 2020b). Collectively, this protein pool is usually referred to as the *myofibrillar fraction* of muscle tissue. Spacing between myofibrils is sparse (i.e., ∼15% of intracellular space is unoccupied by myofibrils according to estimates stated above). These spaces contain organelles and cellular components including mitochondria (likely in the form of a reticulum), the sarcoplasmic reticulum, macromolecules (e.g., ribosomes, glycogen and lipid droplets), and various proteins and enzymes. TEM has been used to show the mitochondria occupy ∼5–6% of the space within muscle fibers, whereas the sarcoplasm occupies ∼9% (Claassen et al., 1989). The aqueous media that myofibrils and non-myofibrillar components reside in is termed the sarcoplasm. Akin to cytoplasm in other cells, the sarcoplasm is critical for maintaining ion and pH balance within muscle fibers. The non-myofibril components of muscle fibers as well as the sarcoplasm make up what has been termed the *sarcoplasmic fraction* of muscle tissue.

**FIGURE 2:**
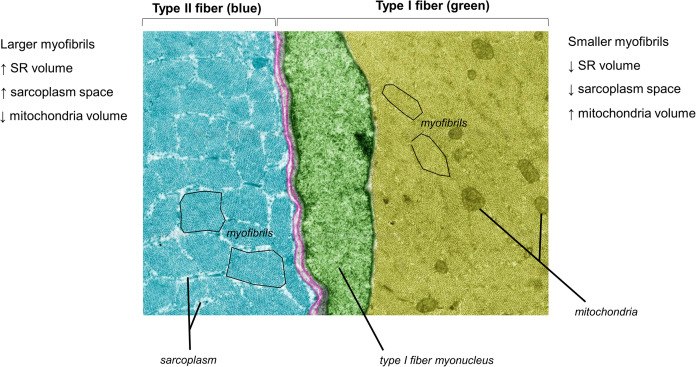
A pseudocolored cross-sectional TEM image from a Wistar rat (8,000×) demonstrating morphological differences between different muscle fiber types. The image was licensed from sciencesource.com (courtesy of Jose Luis Calvo) by the authors for scientific publication purposes.

Figure 1 does not depict how differences in fiber type influence cellular morphology. Animal studies have shown that chief differences between slow-twitch (type I) and fast-twitch (type II) fibers are as follows: (i) slow-twitch fibers contain more mitochondria (or an expanded mitochondrial reticulum) relative to fast-twitch fibers (Schiaffino et al., 1970) (ii) fast-twitch fibers possess larger sarcoplasmic reticulum vesicles surrounding myofibrils (Lee et al., 1991) and (iii) myofibril diameters appear to be slightly larger in fast-twitch fibers, although sarcomeres are slightly longer and z-lines are slightly thicker in slow-twitch fibers (Schiaffino et al., 1970). Some of these fiber type-specific characteristics have also been shown in human skeletal muscle using TEM methods (Alway et al., 1988). Figure 2 is a TEM image that visually demonstrates some of these fiber type-specific differences.

The sarcolemma, or cell membrane of a muscle fiber, is also an important topic of discussion. The sarcolemma contains various proteins including membrane-bound receptors and transporters. The sarcolemma also contains transmembrane proteins, and these proteins form complexes that link intracellular cytoskeletal proteins to proteins that reside in the extracellular matrix (Henderson et al., 2017). Our recent proteomic data indicate that some sarcolemma-associated proteins are enriched in the myofibrillar fraction (e.g., dystrophin), whereas others are enriched in the sarcoplasmic fraction (e.g., vinculin) (Vann et al., 2020b). The process of tissue homogenization likely leads to this differential compartmentalization of sarcolemma-associated proteins. Critically, while these proteins are enriched in one or both fractions, they do not technically fall purely under either classification. This topic will be re-visited later below.

## Methods Used to Assess Sarcoplasmic Hypertrophy

There are four published methods that have been used to report sarcoplasmic hypertrophy may occur in response to resistance training. Three of these methods include TEM, phalloidin staining, and SDS-PAGE and Coomassie staining. The assessment of specific tension in isolated (or skinned) muscle fibers can also provide indirect evidence of sarcoplasmic hypertrophy. The mechanics, advantages, and disadvantages of each method are discussed below.

### TEM

This method involves subjecting a small piece of muscle tissue (∼1–2 mg) to extensive fixative and dehydration steps followed by resin embedding. Nanometer-thick sections are then generated using an ultramicrotome, these sections are placed on small grids, and sections are chemically treated using uranyl acetate and lead citrate to generate contrast between cellular structures. Grids containing one or multiple sections can then be viewed in a TEM. Assuming these methods are performed correctly, images that are yielded from this technique provide myofibrils with good resolution at 1,000–20,000× magnifications. Researchers can then use manual or automated image analysis software to size myofibrils, mitochondria, and the spatial void that exists between these structures (i.e., the sarcoplasm). Myosin and actin filaments can also be imaged at ∼40,000× magnifications in order to examine spacing and lattice-like properties. A major disadvantage of TEM is the technique only provides a two-dimensional image for a portion of one or two muscle fibers. Although multiple images could be randomly acquired across multiple muscle fibers to garner a more holistic representation of the tissue, obtaining 20 images per subject would (at best) only provide data for portions of 20–40 cells. A second limitation to TEM is that fixation, dehydration, embedding, staining, and ultramicrotome methods require days of laboratory work. Not only is this problematic from a throughput perspective, but the high number of steps could artificially alter myofibrillar spacing that would confound measures of sarcoplasmic volume. In this regard, TEM artifacts (e.g., spacing of cellular structures, or shrinkage of organelles) have been reported due to processing errors (Mollenhauer, 1993), and these phenomena may lead to erroneous conclusions about cellular properties. Finally, TEM devices are seldom found in exercise physiology laboratories given the costs and expertise needed to operate and maintain equipment.

### Phalloidin Staining

This is a histological method where a staining solution containing a phalloidin-conjugated fluorophore (e.g., Alexa Fluor 488 Phalloidin; Thermo Fisher, Waltham, MA, United States) is applied to muscle sections adhered to glass microscope slides. The chemical properties of phalloidin enable it to bind with high specificity to actin polymers; thus, one strength of the technique is the specificity of staining. The relative actin abundance in muscle cross-sections incubated with Alexa Fluor 488 Phalloidin can then be obtained in ∼20–30 whole fibers with simple fluorescence microscopy at 200× magnification. If multiple images of a section are obtained across different regions of the tissue then ∼50–100 fibers can be interrogated. With automated pixel counting software, it is then possible to provide an unbiased count of green pixels within dystrophin-stained borders in order to derive the relative abundance of actin per myofiber. Beyond the high binding specificity of phalloidin to actin, another strength of the technique is the sampling of more whole fibers relative to TEM imaging. The steps involved with tissue processing are also less laborious compared to TEM preparation. Like TEM, however, this technique is also limited to a two-dimensional image. Another limitation is that there are non-contractile actin proteins that exist in muscle fibers which serve as scaffolding proteins (Stromer, 1998). While these proteins likely do not make up much of the sarcoplasmic protein fraction, phalloidin likely binds to these proteins and generates signal from non-contractile material. Proper tissue preservation via slow-freezing in optimal cutting temperature media is also necessary to successfully perform this technique. Improper freezing could lead to a commonly observed artifact of large vacuole-like structures, and these structures could be misconstrued as space not occupied by myofibrils (Meng et al., 2014). Readers can refer to our past work if interested in obtaining further details of this method (Haun et al., 2019b). Additionally, Gokhin et al. (2008) have used this method to quantify relative amounts of contractile material per muscle fiber in growing rodents.

### Sodium Dodecyl Sulfate-Polyacrylamide Gel Electrophoresis (SDS-PAGE) With Coomassie Staining

**FIGURE 3:**
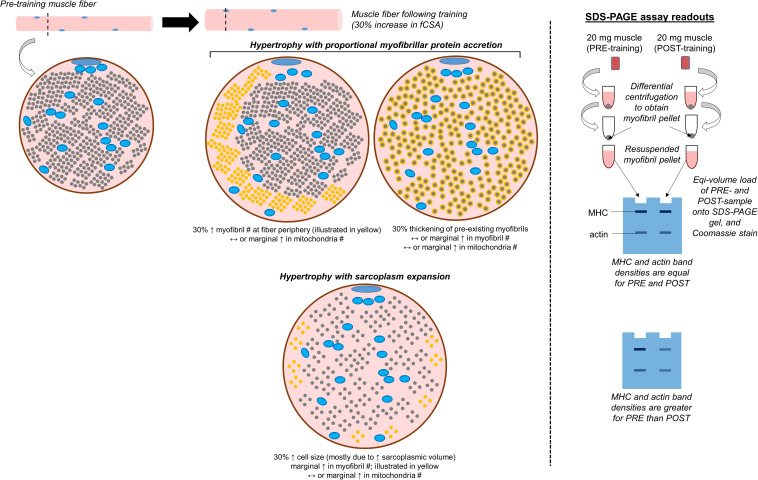
Conceptualization of conventional versus sarcoplasmic hypertrophy, and how these phenomena would manifest with the electrophoresis technique discussed. The left inset demonstrates various modes of muscle fiber hypertrophy. The small blue circles within the fiber represent mitochondria, the smaller gray circles occupying most of the intracellular space represent myofibrils, and the remainder of the space represents the sarcoplasm. In situations where hypertrophy occurs due to the proportional accretion of myofibrillar protein (i.e., conventional hypertrophy), there could either be the addition of new myofibrils to the periphery which “push” the cell outward (indicated as new yellow dots), or there could be the thickening of pre-existing myofibrils (indicated by the haloing of gray circles). Alternatively, sarcoplasmic hypertrophy occurs through the disproportionate expansion of the sarcoplasm relative to myofibril addition. Select studies have shown this process may occur in response to resistance training, although some studies refute this model of hypertrophy. This process could potentially be shown through TEM imaging, phalloidin staining, or the determination of specific tension in isolated fibers (not depicted in figure). This process could also be reflected when analyzing pre- and post-training biopsy specimens through SDS-PAGE and Coomassie staining, and analyzing the changes in myosin heavy chain (MHC) and actin protein band densities (illustrated on the right inset of the figure).

This is a non-histological technique that allows for the determination of the relative abundances of myosin heavy chain, actin, tropomyosin, troponin, and myosin light chain protein isoforms per milligram of wet or dry muscle tissue. Given that we have performed various iterations of this assay (Roberts et al., 2018; Haun et al., 2019b; Vann et al., 2020b), we will provide a more in-depth description of this technique relative to others. We also provide a schematic in Figure 3 which shows how results from the assay are interpreted. It should be noted that other researchers have also performed this technique for the purpose of quantifying the relative amount of contractile protein per unit tissue across various species (Yamada et al., 1997; Thys et al., 2001; Wilkens et al., 2008). Once a muscle biopsy is obtained, the tissue is wrapped in pre-labeled foil, flash-frozen in liquid nitrogen, and transported to −80°C for long-term storage. Given that tissue is frozen in the native state, and muscle fibers contain ∼75% water as stated earlier, this is commonly referred to as *wet tissue*. During the day of experimentation, tissue is removed from −80°C and placed on a liquid nitrogen (LN_2_)-cooled mortar. The tissue is then pulverized with a LN_2_-cooled pestle, and a fraction of the tissue (∼20 mg) is placed in a pre-weighed 1.7 mL tube containing a Tris-based buffer with 0.5% Triton-X 100 and protease inhibitors (termed Buffer 1). These details are critical to note since this first buffer contains the Triton-X detergent which is capable of lysing cell membranes while not solubilizing myofibrils. This buffer may also be one of the reasons that some of sarcolemma proteins are enriched in the sarcoplasmic protein fraction as indicated earlier in the review. Tubes are then re-weighed in order to obtain a wet muscle weight based upon the weight differential between the tube prior to and following the addition of tissue. The tube containing Buffer 1 and tissue is then removed from the analytical scale, pulverized using tight-fitting pestles until a slurry is formed, and tubes are capped and centrifuged thereafter. Following centrifugation, a liquid supernatant and pellet remain. The supernatant contains mostly Buffer 1 as well as sarcoplasmic constituents (e.g., sarcoplasmic proteins, mitochondrial proteins). The pellet contains mostly myofibrillar proteins given that they were not solubilized. However, we have also detected certain histone proteins in the pelleted fraction with proteomics (Vann et al., 2020b). While the presence of these nuclear proteins is minimal relative to other contractile proteins (e.g., myosin heavy chain, titin, nebulin and others), these data suggest the nuclei are likely pelleted during the first centrifugation step. Approximately 90% of the supernatant is removed with a pipette, the supernatant is placed in a new 1.7 mL tube, and this tube containing the sarcoplasmic fraction is stored at −80°C for downstream analyses. Additional Buffer 1 is then added to the original 1.7 mL tube containing the myofibrillar protein pellet, and the pellet is re-suspended to wash the pellet. The resulting slurry is centrifuged, and the resultant supernatant is discarded following centrifugation. The myofibril pellet can be air-dried on ice and then re-suspended in what we have termed Buffer 2, which is a Tris-based buffer containing protease inhibitors as well as potassium chloride, glycerol and spermidine. Notably, all of the latter additives aid in the solubilization of the myofibril pellet. Once myofibrils are re-suspended, a set volume of the resuspension (e.g., 10 μL) can be prepared with reducing buffer and subjected to SDS-PAGE. The gel can then be Coomassie stained, the density of prominent bands existing at ∼220 kD (myosin heavy chain) and 43 kD (actin) can be quantified, and band densities can be divided by muscle weight to obtain relative amounts of myosin heavy chain and actin. In relation to the limitations discussed with TEM and histology, there are advantages to using this method. First, more muscle is sampled relative to TEM or phalloidin staining, and this advantage provides more confidence regarding conclusions made about the physiological state of the tissue. Second, determining the relative abundance (or concentrations) of the two major contractile proteins allows for the extrapolation of the three-dimensional properties of the tissue. Alternatively stated, if the relative abundances of myosin heavy chain and actin decrease with a concomitant increase in tissue area or fCSA during resistance training, then this finding could reflect an increase in sarcoplasm volume across hundreds of muscle fibers. Third, we have shown that this technique exhibits good reproducibility and sensitivity (Roberts et al., 2018). However, as with Western blotting, a major limitation to the method is that it provides data on crude lysates. In this regard, observing training-induced decrements in the relative abundances of myosin heavy chain and actin may indicate localized edema (rather than sarcoplasmic hypertrophy) occurred. Likewise, this method generates a binary classification of proteins as being either myofibrillar or sarcoplasmic. Indeed, our proteomics data has shown that most of the myofibrillar proteins yielded from this method are contractile or other proteins associated with myofibrils, while most of the sarcoplasmic proteins yielded are enzymes that reside in the sarcoplasm (Vann et al., 2020b). However, there are trace amounts of proteins found in each fraction that could be considered contaminants. As mentioned above, we have identified nuclear protein in the myofibrillar fraction, and sarcolemma proteins have been found in both fractions. We have also detected trace amounts of laminin and collagen isoforms in the myofibril fraction. While not a limitation, the researcher should also ensure muscle tissue is visibly free of blood, fat, and connective tissue immediately following the biopsy and prior to snap freezing. In this regard, tissue frozen with residual blood will artificially elevate wet muscle weights, and this technical error will lead to the false conclusion that the relative abundances of contractile proteins are lower when compared to tissue samples that are visibly free of blood. Finally, the suspension of myofibrils in Buffer 2 (described above) is a technical challenge. While we have developed a technique which solubilizes myofibrils (Roberts et al., 2020) researchers interested in utilizing this technique should perform extensive piloting to ensure proper solubilization is optimized prior to scaling up experimentation.

### Specific Tension Assessment in Individual Muscle Fibers

This technique is performed using a specialized force transducer, and can provide an indirect assessment of contractile material per muscle fiber cross-section (D’antona et al., 2007; Degens et al., 2010; Meijer et al., 2015). Readers interested in observing this technique can refer to an excellent visual methods article by Roche et al. (2015), as well as a comprehensive review on the topic by Canepari et al. (2010). This method first involves obtaining a biopsy, cryopreserving the tissue, and freezing it until the day of experimentation. The day of experimentation, tissue is thawed and permeabilized in a solution containing Triton X-100. Tissue is then placed in relaxing solution, individual fibers are dissected out (or skinned), and one fiber is suspended end-to-end to opposing pins with nylon sutures which interface with a force transducer. The tautness of the fiber is adjusted in relaxing solution under a microscope order to achieve an optimal sarcomere length (∼2.6 μm). At this point, fiber diameters can be obtained to extrapolate fCSA values under the presumption skinned fibers adopt a circular cross-section. Fibers are then transferred to a physiological “activation” solution (pH 7.0) containing calcium, ATP and other constituents. The isometric force developed by the fiber is then monitored until a plateau is reached, and this value can be divided by fCSA to derive fiber area-normalized tension (i.e., specific tension). As an additional step, researchers can determine fiber type by removing the fiber from the force transducer apparatus, homogenizing it in specific buffers, performing SDS-PAGE with Coomassie or silver staining, and observing the myosin heavy chain isotype banding pattern. If one were to perform this technique with two fibers that show similar diameters, it can be reasonably concluded that the fiber presenting a lower specific tension value possesses less myofibrils in cross section. The strength of the technique is functional data acquired on fibers *ex vivo*. As a contextual example, it can be challenging to disentangle the role neural adaptations play in the adaptive process when an individual exhibits increases in muscle strength and hypertrophy during a period of resistance training. To this end, single fiber interrogations can provide clearer evidence as to whether muscle fiber hypertrophy and, more specifically, the accretion of myofibril proteins may have contributed to strength increases. A limitation to this technique is that only segments of single fibers are assayed rather than whole-length fibers. Another limitation is that permeabilization alters the intrinsic characteristics of fibers such as calcium sensitivity and osmotic balance, and fibers artificially expand due to these changes as evidenced by appreciably larger fCSA values when compared to fCSA values obtained via histology. A third, and significant, limitation to this technique includes the delicate and time-consuming steps involved with microdissections as well as the delicate nature of interfacing fibers with the force transducer. Because of the work required to perform this technique, it is common for less than 10 fibers per subject to be analyzed (Meijer et al., 2015). Finally, as with TEM, this technique is not widely performed due to the specialized equipment and expertise needed.

Notably, all of the methods discussed above require obtaining skeletal muscle biopsies from human participants which, itself, can be a limitation for many laboratories. Vastus lateralis biopsy sampling is also limited to low tissue yields (∼100 mg) relative to muscle size. In this regard, cadaver analyses show the mass of the entire vastus lateralis muscle averages ∼375 g in humans over 80 years old (Ward et al., 2009), and these masses likely average over 500 g in younger, healthy participants. Thus, a biopsy would sample only 1/2500th of one quadriceps muscle. This figure becomes more paltry when considering TEM interrogates 1–2 mg of tissue, skinned fiber studies interrogate ∼10 of the hundreds of thousands vastus lateralis muscle fibers, histology studies examine dozens of fibers, and biochemical studies interrogate 20–30 mg of tissue. Another limitation with the biopsy technique is the same bundle of fibers are presumably not sampled when performing the method at pre- and post-intervention time points. The unfamiliar reader should be aware of these limitations in order to better appreciate arguments put forth in this review.

## Evidence in Humans That Supports Resistance Training-Induced Sarcoplasmic Hypertrophy

Macdougall et al. (1982) published the first study in humans providing good evidence to suggest sarcoplasmic hypertrophy may appreciably contribute to muscle fiber hypertrophy. Around a century earlier, Morpurgo reported results from the first training-induced hypertrophy study in an animal model, and the author believed fiber size increases were primarily related to the expansion of non-myofibrillar components (Morpurgo, 1897). An earlier report of potential sarcoplasmic hypertrophy with resistance training was also reported in humans by Penman who used TEM methods (Penman, 1969). However, Morpurgo’s study was in animals that treadmill trained, and Penman’s study was limited to 2–3 participants which precluded formal statistical evaluations. Macdougall et al. (1982) examined five participants who engaged in 6 months of upper-arm resistance training. Biopsies were obtained from the triceps muscle prior to and following the training intervention. Muscle samples were analyzed for morphology using TEM methods. Type II fCSA values increased as a result of training, and the authors additionally observed ∼3% decrease in the two-dimensional space occupied by myofibrils (*p* < 0.05) as well as a ∼15% increase in space occupied by the sarcoplasm (*p* < 0.05). More compelling are data in the same paper from seven participants who possessed years of resistance training experience. These individuals possessed larger type II muscle fibers compared to untrained participants prior to the 6-month training intervention, but showed 30% lower values in the space occupied by myofibrils and two-fold greater values in space occupied by sarcoplasm. Toth et al. (2012) used TEM to report that 18 weeks of resistance training decreased space occupied by myofibrils ∼15% in vastus lateralis muscle fibers from healthy individuals. Meijer et al. (2015) subsequently employed isolated fiber techniques and reported that body-builders, who possessed large vastus lateralis muscle fibers, presented specific tension values that were ∼40% lower than untrained participants. Our group used SDS-PAGE and Coomassie staining to determine 6 weeks of very high volume resistance training reduced relative abundances (per milligram dry tissue) of myosin heavy chain and actin by ∼30% in vastus lateralis muscle from 15 well-trained participants (Haun et al., 2019b). Phalloidin staining was also used to show actin protein density per fiber decreased in these participants from pre- to post-intervention. This study was limited given the post-training biopsy was collected after only 24 h of recovery from the last training bout. Notwithstanding, eight of these participants gave a third biopsy 8 days following the last training bout, and features of sarcoplasmic hypertrophy were still evident. More recently, we published a study in 15 previously trained college-aged men where vastus lateralis biopsies were obtained prior to a 10-week training intervention and 72 h following the last training bout (Vann et al., 2020a). Compared to all of the aforementioned studies, participants performed higher load resistance training where exercises were executed using 3–5 sets of 3–8 repetitions at ∼80–90% maximum strength (1RM). On average, type I fCSA values did not change and type II fCSA values increased 19% (*p* < 0.05). While SDS-PAGE and Coomassie staining indicated training reduced the relative abundances of myosin heavy chain and actin protein (per milligram dry tissue) by only ∼3%, these pre- to post-training changes were statistically significant (*p* < 0.05). Notably, these contractile protein decrements per milligram tissue were not nearly as robust as observed in our prior study by Haun et al. (2019b) (i.e., ∼30% decreases). In lieu of the type II fiber hypertrophy observed in our second study, these data suggest appreciable myofibrillar protein accretion likely occurred with short-term higher load training. Notwithstanding, studies from our laboratory suggest sarcoplasmic hypertrophy may be evident with novel training paradigms in previously trained individuals. Additionally, our data imply this mode of hypertrophy occurs more so with higher volume training.

Collectively, the seven human studies mentioned above provide reasonable evidence to suggest sarcoplasmic hypertrophy may occur during resistance training. While all of the studies differed with regard to training duration and load schemes, only our most recent study implemented what would commonly be considered higher-load training (i.e., performing less than five repetitions per set at >85% one repetition maximum). This point is important to note as it relates to content discussed later in the review.

## Evidence in Humans That Refutes Resistance Training-Induced Sarcoplasmic Hypertrophy

Prior to discussing human data which refutes sarcoplasmic hypertrophy being involved with fiber growth, it is important to discuss a pivotal rodent study which preceded the human work. Goldspink (1964) published a report involving four groups of female mice. Two groups were trained to obtain food by operating a resistance-loaded pulley apparatus which targeted the biceps brachii muscle, and the only variable differentiating these groups was the amount of food administered per day (i.e., 3.5 g/d and 5.0 g/d). The other two groups were not housed with pulley devices and served as feed-matched control groups. Following 25 days of this experimental setup, all mice were euthanized and biceps muscles were histologically examined using light microscopy at 1000× magnification. Regardless of food amount administered, mice housed with pulley systems possessed fibers that were 30% larger compared to control animals. A strong linear relationship was also noted between mean fCSA and myofibril number in mice housed with pulley systems. Large, and presumably hypertrophied, fibers in exercised mice also possessed less space occupied by sarcoplasm relative to smaller fibers. In explaining his findings, the author stated (p. 215):

It was shown that the number of myofibrils per fiber increases in a linear manner with increase in the diameter of the fiber. This means that the increase in the number of [myo]fibrils does not proceed at the same rate as the increase in the cross sectional area of the fiber. It was not possible to accurately measure the diameter of [myo]fibrils using light microscopy but it seems most likely that they increase in girth with increase in fiber size.

While limited to rodents, these data suggest an appreciable amount of myofibril protein accretion accompanied resistance load-induced muscle fiber hypertrophy. Moreover, Goldspink’s data suggest myofibrils increase in girth rather than number, and a slight degree of *myofibril packing* – or a disproportionate increase in myofibril protein accretion relative to fiber growth – may be involved with load-induced fiber hypertrophy. Goldspink later authored an excellent book chapter discussing the mechanisms of myofibril addition with fiber growth during periods of exercise training (Goldspink, 2011). He cited numerous animal studies, many which were performed by Goldspink (2011), suggesting: (i) the creation of new myofibrils (i.e., *de novo* myofibrillogenesis) only occurs during embryonic development, (ii) myofibril protein accretion in sexually mature animals likely involves newly synthesized proteins being added to the periphery of currently existing myofibrils, and (iii) myofibrils can proliferate during periods of exercise training, although this is likely due to myofibril splitting.

While Goldspink’s work has added tremendous knowledge regarding the behavior of myofibrils, no human studies to date have provided this level of detail. In agreement with Goldspink’s findings, however, are sparse human data that exist which refute the notion that resistance training-induced sarcoplasmic hypertrophy precedes or contributes to fiber or tissue growth. Luthi et al. (1986) used TEM to examine morphological adaptations in vastus lateralis muscle fibers from eight untrained college-aged men that resistance trained for 6 weeks. While vastus lateralis area increased by 8.4%, fCSA values and the density of myofibrils remained unchanged. These findings suggest that the proportional accretion of myofibril protein accompanied muscle growth during training. TEM data was also combined with mid-thigh computed tomography data to estimate that the absolute volume occupied by myofibrils increased ∼10% across the entirety of leg extensor muscles. Critically, what was not delineated was whether this increase in myofibril content occurred through the creation of new myofibrils, the enlargement of pre-existing myofibrils, and/or myofibril splitting. Moreover, assuming the morphology acquired through TEM applies to all leg extensor muscles has limitations given that TEM examines an exceedingly small amount of tissue as discussed above. Finally, the lack of fCSA increases with training in lieu of muscle area increases is difficult to reconcile, and speaks to a larger issue of the lack of agreement between methods used to assess hypertrophy as we have previously discussed (Haun et al., 2019c). A follow-up study by this same research group examined high magnification TEM images from the same participants to determine changes in thick and thin filament spacing with training (Claassen et al., 1989). Spacing between myosin filaments was similar at the pre- and post-intervention time points. These findings provide further evidence that myofibril protein accretion was proportional to tissue growth. Previously obtained computed tomography data was also used in the latter report to show that the radiological density of the mid-thigh muscles increased with training. Interestingly, the researchers attributed this latter finding to a potential increase in myofibril packing. Although a reasonable conclusion, this finding may have also been related to a decrease in intramuscular lipid or an increase in non-contractile protein content (e.g., extracellular matrix proteins). Further, the authors’ presumption that myofibril packing occurred conflicts with their prior TEM data suggesting that, while myofibril content increased in absolute terms, myofibril density and spacing were preserved. A study by Trappe et al. (2000) examined specific tension changes in isolated vastus lateralis muscle fibers from seven previously untrained older men after 12 weeks of resistance training. Although type I and IIa fibers exhibited 20% and 13% increases in fCSA values, specific tension values in both fiber types were similar at the pre and post time points. Similar to the report by Luthi et al. (1986), these results suggest a proportional accretion of myofibril protein accompanied fiber growth. Trappe’s group published a follow-up study in seven older, untrained females who engaged in 12 weeks of resistance training (Trappe et al., 2001). Unlike males in their prior report, type IIa fibers did not hypertrophy. Additionally, statistical comparisons indicated specific tension values in both fiber types were not affected with training. These findings suggest training did not affect type IIa fiber morphology, and radial growth in type I fibers was accompanied by a proportional accretion of myofibril protein. Similar findings have been reported by other researchers in six previously untrained men that underwent 12 weeks of resistance training (Widrick et al., 2002); specifically, vastus lateralis muscle fiber hypertrophy occurred without alterations in isolated fiber specific tension values.

More recently, our group examined vastus lateralis tissue adaptations that occurred in previously untrained college-age males who underwent 12 weeks of resistance training (Roberts et al., 2018). Participants were clustered into high and low responders based on a composite score of pre- to post-intervention changes in mean fCSA, dual energy X-ray absorptiometry-determined total body lean tissue mass, and vastus lateralis thickness. The 13 high responders experienced a 34% increase in mean fCSA and 24% increase in vastus lateralis thickness. However, these individuals experienced no changes in myosin heavy chain and actin protein abundances per milligram wet tissue, and the same was found for the 12 low anabolic responders who exhibited virtually no change in mean fCSA. Similar to many of the studies above, our findings suggest myofibril protein accretion was proportional to fiber and tissue growth in the high responders. Collectively, these studies largely suggest muscle fiber or tissue growth in response to 6–12 weeks of resistance training occurs through conventional rather than sarcoplasmic hypertrophy. In relation to hypotheses posited later in the review, it is important to note that all of these studies examined previously untrained participants.

Various tracer studies also question the veracity of sarcoplasmic hypertrophy. For instance, Moore et al. (2009) reported that one bout of resistance exercise elicits robust increases in fasting myofibrillar protein synthesis rates and minimal alterations in sarcoplasmic protein synthesis rates within a 5-h post-exercise window. Further, these authors reported that sarcoplasmic protein synthesis rates were most responsive to post-exercise nutrient provision rather than exercise itself. Gasier et al. (2012) published similar findings using the D_2_O tracer method to show that one bout of lower body resistance exercise stimulated an increase in myofibrillar protein synthesis rates while not affecting overall protein synthesis rates over a 16-h post-exercise window. Wilkinson et al. (2014) also used the D_2_O tracer method to show that 4 days of unilateral leg resistance training over an 8-day period significantly increased myofibrillar protein synthesis rates while not affecting sarcoplasmic protein synthesis rates. Similarly, others have used the D_2_O tracer method to show that myofibrillar protein synthesis rates are elevated weeks following the initiation of resistance training (Brook et al., 2015). These data collectively suggest that the myofibrillar protein pool, rather than the sarcoplasmic protein pool, may experience the most robust expansion at the onset of training. However, again, all of these studies were in untrained participants.

Aside from these shorter-term interventions, there are also chronic interventions and cross-sectional comparisons that legitimately question whether sarcoplasmic hypertrophy is involved with long-term muscle hypertrophy. Alway et al. (1988) used TEM to report that intracellular space occupied by myofibrils in gastrocnemius muscle fibers were similar between resistance-trained, endurance-trained, and sedentary subjects despite fCSA values being greatest in resistance-trained subjects. Shoepe et al. (2003) compared vastus lateralis fiber characteristics from six untrained versus six well-trained college-aged men (self-reported training age averaged ∼7 years). While type I and II fCSA values were significantly greater in the well-trained cohort, specific tension values were similar between groups suggestive of long-term fiber hypertrophy with a proportional accretion of myofibril protein. The two aforementioned studies suggest long-term fiber growth with resistance training occurs through conventional hypertrophy. However, these data conflict with other reports. Pansarasa et al. (2009) examined vastus lateralis fiber characteristics in five college-aged women that resistance trained for 1 year. Specific tension significantly increased in type I and II fibers, whereas mean fCSA values demonstrated no change from pre- to post-intervention. Although difficult to reconcile, these findings support a model of longer-term hypertrophy where myofibril packing occurred without increases in muscle fiber hypertrophy. Our group recently examined vastus lateralis thickness and tissue characteristics in six well-trained college-aged men (self-reported training age averaged ∼10 years) versus six untrained men (Vann et al., 2020b). While muscle thickness was 22% greater (*p* < 0.05) in the trained cohort, the abundances of myosin heavy chain and actin per milligram wet muscle were also ∼9% greater (*p* < 0.05). Type I and II fCSA values were numerically greater, but statistically similar between cohorts. D’Antona et al. (2006) also reported that individual type II fiber specific tension values were significantly greater in well-trained bodybuilders compared to recreationally trained controls. Again, findings from both studies suggest long-term hypertrophy is accompanied by myofibril packing. Contrary to the implications from all of the aforementioned studies are two studies in well-trained body-builders suggesting features of sarcoplasmic hypertrophy are evident (Macdougall et al., 1982; Meijer et al., 2015). Collectively, these discordant findings make it difficult to determine how long-term resistance training affects myofibril density and/or if sarcoplasmic hypertrophy is an involved process as individuals become more trained. It is plausible that different doses of resistance training throughout years of training could elicit differential changes in muscle morphology. This is an unresolved area and more research is required to better understand adaptive outcomes at the cellular level in response to different styles of training.

## Putting Forth a Paradigm Considering the Collective Evidence

Considering the aforementioned literature, it is difficult to construct a collective paradigm of muscle fiber growth involving sarcoplasmic hypertrophy. Nonetheless, we speculate there are three candidate scenarios where sarcoplasmic hypertrophy could manifest with resistance training. Specifically, we posit sarcoplasmic hypertrophy is either: (i) a transient symptom of training-induced edema, (ii) a transient mechanism for muscle fiber growth, and/or (iii) an outcome of a myofibrillar protein accretion threshold being reached in well-trained individuals.

Tissue edema is associated with localized damage, and it manifests as a retention of interstitial fluid between cells. Specific to exercise-induced muscle damage, edema can be accompanied by sarcolemma damage where an infiltration of large serum proteins (e.g., albumin) and fluid occurs within and around muscle fibers (Valle et al., 2013). Under these circumstances, it seems plausible edema could artificially elevate fCSA values through intracellular fluid retention, and this would manifest as an expansion of the sarcoplasm. In support of this hypothesis, Yu et al. (2013) examined how extensive downstairs running affected soleus muscle mean fCSA in college-aged men. This study was limited in that repeat biopsies were not obtained from participants; specifically, 16 men performed the exercise bout, and biopsies were obtained from a subset of participants at 1 h post-exercise, 2–3 days post-exercise, and 7–8 days post-exercise. This study was also limited in that high volume downstairs running has little resemblance to resistance exercise. Notwithstanding, mean fCSA values were ∼30% greater in biopsies obtained 7–8 days post-exercise versus biopsies obtained at the earlier time points, and the authors posited this was primarily associated with localized edema. While informative, it is difficult to determine if sarcoplasmic hypertrophy was evident given that ultrastructural interrogations were not performed. Nonetheless, this study demonstrates fCSA changes days following muscle damage is due to edema, and this is likely manifested through an expansion of the sarcoplasm.

Non-invasive assessments of edema can also be performed by capturing muscle images using ultrasound or magnetic resonance imaging (MRI) and quantifying B-mode echo intensity or signal intensity in T2-weighted images, respectively. Damas et al. (2016c) monitored changes in vastus lateralis B-mode echo intensity in untrained college-aged men prior to, 3 weeks into, and following a 10-week resistance training intervention. While increases in muscle area were reported at the 3-week and post-training time points, increased edema (i.e., B-mode signal intensity/vastus lateralis area) was only found to occur at the 3-week time point. The authors concluded muscle hypertrophy was confounded by edema 3 weeks, but not 10 weeks, into training. This paper also led to discussions as to whether muscle hypertrophy could be accurately assessed without the confounding effects of edema within the first month of training in previously untrained participants (Damas et al., 2016b; Defreitas et al., 2016). When relating these findings to the notion of training-induced sarcoplasmic hypertrophy, it is plausible this phenomenon could likely be observed in either: (i) untrained individuals during the first month of training, or (ii) trained individuals exposed to unaccustomed training paradigms. Work from our laboratory by Haun et al. (2019b) which demonstrated training facilitated aspects of sarcoplasmic hypertrophy aligns with the latter hypothesis since participants, while well-trained, performed extraordinarily high and unaccustomed training volumes. Further supporting this contention are prior data in these participants showing increases in whole-body extracellular fluid content (assessed using bioelectrical impedance spectroscopy, or BIS) significantly contributed to increases in whole-body muscle mass changes with training (Haun et al., 2018). While we previously posited edema did not confound outcome variables in this study (Haun et al., 2019b), the aforementioned BIS data does provide evidence to the contrary. Thus, moving forward, researchers should consider the confounding effects of edema when measuring gross surrogates of muscle hypertrophy as well as morphological or biochemical changes in muscle tissue. Moreover, the influence of hydration status on macro- and micro- variables related to hypertrophy should continue to be explored given that fluid shifts have been reported during chronic periods of resistance training (i.e., intramuscular water and plasma volume) (Reidy et al., 2017).

**FIGURE 4:**
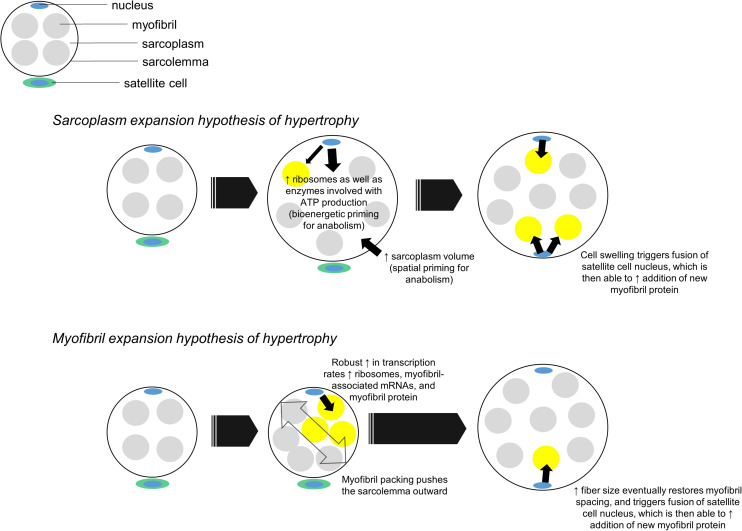
Two hypothetical mechanisms of muscle fiber hypertrophy. In our proposed *sarcoplasm expansion hypothesis of hypertrophy* model, muscle fibers first grow through sarcoplasmic hypertrophy. This allows resident myonuclear domains to spatially, bioenergetically, and biosynthetically prepare for the accretion of additional myofibrils. Although resident domains may synthesize and accumulate some myofibril protein during this phase of hypertrophy, we hypothesize the predominance of sarcoplasmic hypertrophy reduces the transcriptional burden on resident domains. Once sarcoplasmic hypertrophy leads to appreciable cell growth, an additional myonuclear domain is acquired via satellite cell fusion. This new myonuclear domain can then appreciably contribute to myofibril protein accretion by “backfilling” space created through sarcoplasm expansion. In the *myofibril expansion hypothesis of hypertrophy* model, muscle fibers rapidly synthesize and accrue myofibril protein. This phenomenon leads to myofibril packing which acts to push the sarcolemma outward. Fibers eventually reach a size threshold, which leads to an additional myonuclear domain being acquired via satellite cell fusion. At this point both domains can operate to maintain and further synthesize new myofibrils.

Aside from being a potential symptom of edema, we propose sarcoplasmic hypertrophy may be a mechanism that contributes to the early phases of radial fiber growth. It is common for mean fCSA to increase by ∼15–30% in response to months of resistance training (reviewed in Grgic and Schoenfeld, 2018). These findings suggest muscle fibers need to generate an appreciable amount of intracellular space to accumulate more myofibril protein. One potential mechanism to accomplish such growth could involve sarcoplasmic hypertrophy followed by “backfilling” of newly generated sarcoplasm space with myofibrillar protein. This mechanism contrasts a notion put forth by Phillips (Phillips, 2000) where myofibril packing precedes hypertrophy and newly added myofibril material “pushes” the sarcolemma outward. While neither mechanism has definitive supporting evidence in humans, evidence in other cell lines suggests sarcoplasmic hypertrophy may be favored during muscle fiber growth. In this regard, Neurohr et al. (2019) recently demonstrated cytoplasmic expansion readily occurs in yeast and primary mammalian cells through the disruption of one gene (*Cdc28*). The authors also noted RNA and protein biosynthesis did not scale with increased cell growth, and hypothesized this phenomena was due to the transcriptional burden placed on nuclei. This work is limited given that cell growth in intact organisms is constrained by connective tissue, whereas this constraint is not applicable in monolayer culture work. Nonetheless, these results demonstrate in principle that cytoplasmic expansion is a conserved mechanism of cellular hypertrophy in yeast and across numerous mammalian cells. The data by Neurohr et al. (2019) also reiterate the notion cells possess an optimal DNA: cell volume ratio since the processes of ribosome, mRNA and protein biosynthesis are resource-intensive (reviewed in Gillooly et al., 2015). Although muscle fibers are multinucleated, the myonuclear domain theory operates under a similar presumption (Allen et al., 1999) specifically, an increase in muscle fiber size requires the addition of myonuclei from satellite cells in order to support the accretion and maintenance of newly added intracellular material. Thus, muscle fibers may preferentially grow through sarcoplasmic hypertrophy early into a training intervention given: (i) this mechanism appears to be conserved across several cell types as discussed above, (ii) myonuclear accretion lags early into training, and some studies have reported fiber growth occurs without myonuclear accretion (reviewed in Murach et al., 2018), and (iii) if fiber growth occurred through conventional hypertrophy or myofibril packing without myonuclear accretion, then resident myonuclei would likely be stressed in coordinating the large-scale production of ribosomes, mRNAs that encode large myofibril proteins, and additional mRNAs needed for cellular homeostasis. This concept is further illustrated in Figure 4.

In relation to Figure 4, our proposed sarcoplasm expansion hypothesis of muscle hypertrophy during resistance training involves the following:

(i) Prior to satellite cell-mediated myonuclear addition, muscle fibers undergo spatial priming via sarcoplasmic hypertrophy, bioenergetic priming via an up-regulation in enzymes needed for ATP generation, and protein biosynthesis priming via ribosome biogenesis. These phenomena spatially, “bioenergetically,” and “biosynthetically” prepare fibers for myofibril protein accretion while effectively managing the transcriptional burden on resident myonuclei.
(ii) Once satellite cell-mediated myonuclear addition occurs, new myonuclear domains can facilitate a significant upregulation in the synthesis of myofibril-associated mRNAs and myofibril proteins. This is the backfilling of sarcoplasm space with contractile components.

In relation to the phenomena discussed above, it is notable participants who demonstrated signatures of sarcoplasmic hypertrophy in our higher volume training study did not exhibit increases in myonuclei counts per fiber (Haun et al., 2019a, b). Thus, while speculative, a reason why sarcoplasmic hypertrophy may have occurred could be related to the transcriptional burden placed on resident myonuclei without myonuclear accretion as discussed above. These participants also demonstrated increases in ribosome biogenesis as well as increases in the protein expression of sarcoplasmic enzymes responsible for generating ATP through the ATP-PCr and glycolytic pathways. This latter observation could be related to the protein biosynthesis and bioenergetic priming aspects of sarcoplasmic hypertrophy discussed above. While these data are compelling, more research is needed to validate this model.

**FIGURE 5:**
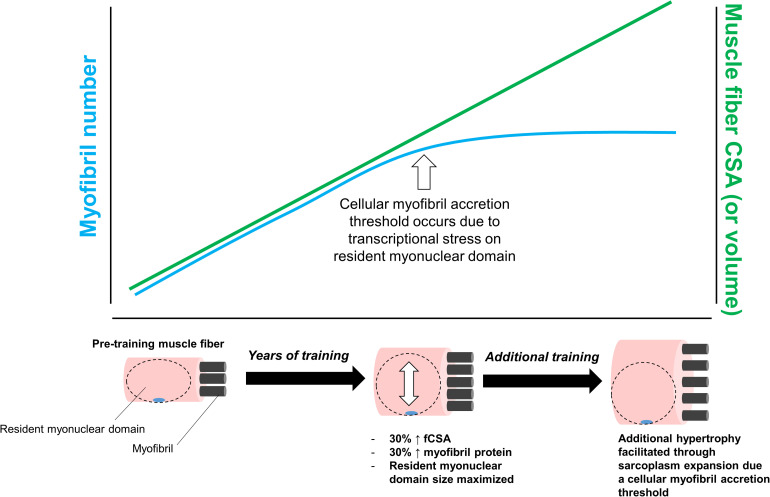
This figure puts forth the hypothesis that sarcoplasmic hypertrophy with years of resistance training may be due to large muscle fibers exceeding a myofibril accretion threshold. This phenomenon is similar to the transcriptional stress theory posited in Figure 3. Specifically, we propose muscle cells grow via myofibril accretion due to a certain threshold size. Thereafter, fibers can no longer add myofibrils due to significant stress to resident myonuclear domains. Based on limited evidence cited in-text, we propose this mechanism may be evident in individuals who engage in higher volume training for years (e.g., bodybuilders).

Finally, we posit that a third scenario involving sarcoplasmic hypertrophy may involve a myofibril protein accretion threshold being reached in large muscle fibers with years of resistance training. We posit this mode of hypertrophy would occur for similar reasons discussed above; specifically, transcriptional stress imposed on resident myonuclei in large muscle fibers would favor cellular growth through sarcoplasmic hypertrophy. This hypothesis is further illustrated in Figure 5 below.

Again, this hypothesis is speculative given that, as with the two other aforementioned hypotheses, there is virtually no supporting data. Additionally, it is currently unknown as to whether a myofibril accretion threshold exists in muscle fibers. In support of this hypothesis, however, other researchers have theorized a protein accretion threshold occurs in mitotically active cells, and this threshold acts to trigger cellular division (Soltani et al., 2016). Since myofibers are post-mitotic cells that cannot divide, sarcoplasmic hypertrophy may be the only manner through which large fibers are capable of growing. Indeed, this is a potential explanation as to why some of the aforementioned studies have reported features of sarcoplasmic hypertrophy in body-builders with years of training experience (Macdougall et al., 1982; Meijer et al., 2015). This also may be why we have observed features of sarcoplasmic hypertrophy in previously trained individuals who underwent novel training paradigms (Haun et al., 2019b; Vann et al., 2020a). As an aside, another theory has been put forth in explaining how large muscle fibers behave in response to hypertrophic stimuli. Murach et al. (2019) have posited mammalian muscle fibers (and myofibrils within fibers) are capable of splitting in response to overload, and there are human and animal data provided by the authors which support these hypotheses [examples include references (Goldspink, 1970; Timson et al., 1985; Antonio and Gonyea, 1994)]. Whether a myofibril protein accretion threshold within a muscle fiber triggers fiber splitting remains unknown. Moreover, beyond two-dimensional analysis on serial sections via histology (Eriksson et al., 2005, 2006), fiber splitting in humans has not been convincingly demonstrated. Other researchers have also provided compelling evidence to suggest perceived fiber splitting in rodent muscle subjected to overload is primarily due to alterations in pennation angle and fiber length rather than splitting (Jorgenson and Hornberger, 2019). Clearly there is still much to be resolved regarding the physiological response of large myofibers to hypertrophic stimuli.

While the synergist ablation model in rodents has its limitations (e.g., rapid and non-physiological muscle growth in response to a constant growth stimulus), such rodent experiments may yield insightful information to support or refute the theoretical mechanisms proposed above. Notably, researchers have already given thought to transcriptional stress elicited on resident myonuclei during overload-induced hypertrophy. McCarthy’s group (Kirby et al., 2016) administered 5-ethynyl uridine to wild-type mice with the intent of labeling nascent RNA transcription in response to overload-induced hypertrophy. These authors examined multiple groups of mice including sham-operated controls (CTL) or mice exposed to three, seven, or 14 days of plantaris overload via synergist ablation (SA). There was a linear increase in plantaris weights across groups where CTL < 3-day SA < 7-day SA < 14-day SA. However, nascent transcription was only up-regulated in the 3-day SA versus CTL mice. We interpret these data to suggest that, while resident myonuclei exhibit a transcriptional reserve during hypertrophy, this response is rapid, pulsatile, and limited in duration. Critically, it was also reported that nascent transcription was lowest in the largest fibers during SA. This finding resonates with those of Neurohr et al. (2019) who showed RNA biosynthesis does not scale in mammalian cells that grow through cytoplasmic hypertrophy *in vitro*. Whether or not the findings of Kirby et al. (2016) indicate large, hypertrophying fibers predominantly grow through sarcoplasmic hypertrophy remains to be determined. Nonetheless, we find these parallels between studies interesting and deserving of further interrogation. We also posit the Pax7-DTA mouse model could be informative in determining whether sarcoplasmic hypertrophy accompanies fiber growth during the early phases of overload-induced hypertrophy. Pax7-DTA mice are genetically modified so that, when administered tamoxifen, these mice show almost a complete ablation of satellite cells due to *Cre-ER*-mediated events that drive the expression of diphtheria toxin fragment A (DTA) in Pax7+ cells (Lepper et al., 2011). Mccarthy et al. (2011) have shown that, while myonuclear accretion is abrogated in these mice during synergist ablation-induced plantaris hypertrophy, fiber growth was not impaired. If our hypotheses above were true regarding resident myonuclei being stressed during overload-induced fiber growth, then it is possible sarcoplasmic hypertrophy occurred more so in mice administered tamoxifen (i.e., those showing no myonuclear accretion) versus mice that were not administered tamoxifen (i.e., those showing myonuclear accretion). However, muscle morphology was not assessed in these animals. Likewise, others have shown that plantaris fiber growth in response to synergist ablation is blunted in Pax7-DTA mice administered tamoxifen (Egner et al., 2016). Given our hypotheses as well as these conflicting data on the Pax7-DTA model, future experiments examining the morphological adaptations that occur in this mouse model are warranted.

## Broader Implications

The topic of sarcoplasmic hypertrophy has existed in the athletic sphere for decades. Stone mentioned this as being a potential training adaptation in the early 1980s (Stone et al., 1983) and Kraemer and Zatsiorsky (2006) speculated conventional versus sarcoplasmic hypertrophy result from high and low-load training, respectively (Kraemer and Zatsiorsky, 2006). While these hypotheses are speculative to a certain extent, there are data to support volume-load paradigms affecting myofibrillar density. In this regard, two of the studies mentioned above which have observed features of sarcoplasmic hypertrophy have been in well-trained bodybuilders (Macdougall et al., 1982; Meijer et al., 2015), and this population generally trains with higher volumes. Conversely, there are features of myofibril packing in power athletes (Meijer et al., 2015; Fluck et al., 2019), and this population generally trains with higher loads and lower volumes. The two aforementioned studies from our laboratory also support this model (Haun et al., 2019b; Vann et al., 2020a), and it is notable that a reduction in myofilament packing density via sarcoplasmic hypertrophy may also promote certain training adaptations such as increased muscle fiber shortening velocity (Metzger and Moss, 1987).

The mainstream relevance of sarcoplasmic hypertrophy with resistance training is also gaining momentum. Since 2014, several online articles have been published for the lay population discussing sarcoplasmic hypertrophy with resistance training. Likewise, the world’s largest online encyclopedia^1^ discusses nuances related to myofibrillar versus sarcoplasmic hypertrophy under a parent article titled *Muscle hypertrophy* despite neither mechanism having been extensively validated. Hence, it is apparent that the general audience is interested in learning more about this aspect of muscle physiology. Moving forward, multiple laboratories will need to pursue this research question in order to validate previous findings and provide meaningful discoveries.

Determining whether sarcoplasmic hypertrophy is a scientific “unicorn” or a resistance training adaptation is paramount for the field for several reasons. To this end, some have posited *true hypertrophy* occurs only when myofibrillar protein accretion is proportional to fCSA increases (Damas et al., 2015; Taber et al., 2019). This is an insightful hypothesis for several reasons. First, others have noted that resistance-training induced skeletal muscle hypertrophy is not strongly associated with strength increases (Ahtiainen et al., 2016) alternatively stated, bigger muscles following training do not yield a proportional increase in strength. This likely holds true for a variety of reasons, many of which have been explained by Taber et al. (2019). To reiterate a key point from Taber et al. (2019), the role sarcoplasmic hypertrophy may have in explaining this imperfect relationship, while small, should be further examined. As also mentioned, some of our research findings suggest high volume training promotes sarcoplasmic hypertrophy to a greater extent compared to high load training. Defining each mode of training is inherently difficult. For instance, some would consider performing sets containing more than 10 repetitions at ∼60–70% 1RM to be higher volume training, whereas others may consider high volume training as performing sets of high repetitions to failure using 30% of 1RM. Relevant to the themes discussed herein are the findings of Mitchell et al. (2012) who reported 80% 1RM and 30% 1RM leg extensor resistance training elicited similar increases in mid-thigh muscle cross-sectional area as well as muscle fCSA. While hypertrophic features were similar with each form of training, investigating how each form of training affects muscle morphology will be insightful given some of our own research findings mentioned above. Other interesting data which may be related to sarcoplasmic hypertrophy comes from Kadi et al. (2004) who obtained serial vastus lateralis biopsies from untrained participants during a 90-day resistance training intervention followed by a 90-day detraining period. Following 90 days of training, mean fCSA increased 16% from pre-training values. Three days of detraining numerically increased fCSA ∼3% more from the 90-day point, and 19% from pre-training. Remarkably, mean fCSA values returned back to pre-training levels after only seven additional days of detraining. In essence, these data suggest a rapid decay in mean fCSA values occurs during detraining. We have contended that, had muscle fibers undergone significant myofibrillar accretion, it would take longer to observe detraining-induced atrophy (Roberts et al., 2018). However, if sarcoplasmic hypertrophy was a significant contributor to fiber growth, then it remains plausible a restoration of the sarcoplasm space to pre-training values may have largely contributed to this detraining phenomenon. Thus, again, utilizing methods to assess cellular morphology in these scenarios will provide useful information in relation to detraining adaptations. Finally, there are long-term resistance training data published by Fatouros et al. (2005) where it is tempting to speculate that some study outcomes may have been due, in part, to sarcoplasmic hypertrophy. These authors divided previously untrained older men into a low-intensity training group (*n* = 18) and high-intensity training group (*n* = 20). Both groups performed whole-body resistance training for 6 months, but the former group utilized weights corresponding to ∼55% 1RM whereas the latter group utilized weights corresponding to ∼82% 1RM. Upper body and lower body strength was measured using the chest press and leg press exercises, respectively, prior to and following training. All participants then detrained for 48 weeks, and strength was re-assessed. Those in the high-intensity group gained more strength with training, which is to be expected. However, those in the high-intensity group maintained more strength compared to those in the low-intensity group following the extensive detraining period. Mid-thigh circumferences and skinfolds were also obtained at all of the aforementioned time points, but no notable differences in muscle growth or atrophy were noted between groups; this is likely due to the relatively poor resolution skinfold and circumference assessments provide. Although these findings may partially be related to long-term neural adaptations with higher load training, it is tempting to speculate higher load resistance training may have also facilitated more myofibril protein accretion compared to lower-load training given the arguments posed above. In this regard, replicating studies like these where biopsies are analyzed for features of conventional versus sarcoplasmic hypertrophy is warranted.

## Conclusion

Research suggests different morphological adaptations may occur with muscle fiber hypertrophy during periods of resistance training. However, there are still significant knowledge gaps in the human literature regarding how different forms of training or training duration affect morphological adaptations. In spite of this paucity of data, our ultimate intent in authoring this review is to spark further interest in the research topic. Such pursuits will confirm or refute whether sarcoplasmic hypertrophy has a role in facilitating resistance training adaptations.

## Author Contributions

MR primarily constructed the review. All authors provided critical edits and approved the manuscript in its current form.

## Conflict of Interest

CH was employed by Fitomics, Inc. during the assembly of this manuscript. The remaining authors declare that the research was conducted in the absence of any commercial or financial relationships that could be construed as a potential conflict of interest.

## Footnotes

1 www.wikipedia.com

## References

[B1] Ahtiainen J. P. Walker S. Peltonen H. Holviala J. Sillanpaa E. Karavirta L. (2016). Heterogeneity in resistance training-induced muscle strength and mass responses in men and women of different ages. *Age* 38:10. 10.1007/s11357-015-9870-1 PMC5005877 26767377

[B2] Allen D. L. Roy R. R. Edgerton V. R. (1999). Myonuclear domains in muscle adaptation and disease. *Muscle Nerve* 22 1350–1360. 10.1002/(sici)1097-4598(199910)22:10<1350::aid-mus3>3.0.co;2-8 10487900

[B3] Alway S. E. Macdougall J. D. Sale D. G. Sutton J. R. Mccomas A. J. (1988). Functional and structural adaptations in skeletal muscle of trained athletes. *J. Appl. Physiol.* 64 1114–1120. 10.1152/jappl.1988.64.3.1114 3366734

[B4] Antonio J. Gonyea W. J. (1994). Muscle fiber splitting in stretch-enlarged avian muscle. *Med. Sci. Sports Exerc.* 26 973–977. 7968431

[B5] Bell K. E. Brook M. S. Snijders T. Kumbhare D. Parise G. Smith K. (2019). Integrated myofibrillar protein synthesis in recovery from unaccustomed and accustomed resistance exercise with and without multi-ingredient supplementation in overweight older men. *Front. Nutr.* 6:40. 10.3389/fnut.2019.00040 31032258 PMC6470195

[B6] Brook M. S. Wilkinson D. J. Mitchell W. K. Lund J. N. Szewczyk N. J. Greenhaff P. L. (2015). Skeletal muscle hypertrophy adaptations predominate in the early stages of resistance exercise training, matching deuterium oxide-derived measures of muscle protein synthesis and mechanistic target of rapamycin complex 1 signaling. *FASEB J.* 29 4485–4496. 10.1096/fj.15-273755 26169934

[B7] Canepari M. Pellegrino M. A. D’antona G. Bottinelli R. (2010). Single muscle fiber properties in aging and disuse. *Scand. J. Med. Sci. Sports* 20 10–19. 10.1111/j.1600-0838.2009.00965.x 19843264

[B8] Claassen H. Gerber C. Hoppeler H. Luthi J. M. Vock P. (1989). Muscle filament spacing and short-term heavy-resistance exercise in humans. *J. Physiol.* 409 491–495. 10.1113/jphysiol.1989.sp017509 2585299 PMC1190456

[B9] Damas F. Phillips S. Vechin F. C. Ugrinowitsch C. (2015). A review of resistance training-induced changes in skeletal muscle protein synthesis and their contribution to hypertrophy. *Sports Med.* 45 801–807. 10.1007/s40279-015-0320-0 25739559

[B10] Damas F. Phillips S. M. Libardi C. A. Vechin F. C. Lixandrao M. E. Jannig P. R. (2016a). Resistance training-induced changes in integrated myofibrillar protein synthesis are related to hypertrophy only after attenuation of muscle damage. *J. Physiol.* 594 5209–5222. 10.1113/jp272472 27219125 PMC5023708

[B11] Damas F. Phillips S. M. Lixandrao M. E. Vechin F. C. Libardi C. A. Roschel H. (2016b). An inability to distinguish edematous swelling from true hypertrophy still prevents a completely accurate interpretation of the time course of muscle hypertrophy. *Eur. J. Appl. Physiol.* 116 445–446. 10.1007/s00421-015-3287-5 26515294

[B12] Damas F. Phillips S. M. Lixandrao M. E. Vechin F. C. Libardi C. A. Roschel H. (2016c). Early resistance training-induced increases in muscle cross-sectional area are concomitant with edema-induced muscle swelling. *Eur. J. Appl. Physiol.* 116 49–56. 10.1007/s00421-015-3243-4 26280652

[B13] D’Antona G. Lanfranconi F. Pellegrino M. A. Brocca L. Adami R. Rossi R. (2006). Skeletal muscle hypertrophy and structure and function of skeletal muscle fibres in male body builders. *J. Physiol.* 570 611–627. 10.1113/jphysiol.2005.101642 16339176 PMC1479884

[B14] D’antona G. Pellegrino M. A. Carlizzi C. N. Bottinelli R. (2007). Deterioration of contractile properties of muscle fibres in elderly subjects is modulated by the level of physical activity. *Eur. J. Appl. Physiol.* 100 603–611. 10.1007/s00421-007-0402-2 17273882

[B15] Defreitas J. M. Beck T. W. Stock M. S. (2016). The findings of Damas et al. have not influenced the previously proposed time course of skeletal muscle hypertrophy. *Eur. J. Appl. Physiol.* 116 443–444. 10.1007/s00421-015-3286-6 26497192

[B16] Degens H. Bosutti A. Gilliver S. F. Slevin M. Van Heijst A. Wust R. C. (2010). Changes in contractile properties of skinned single rat soleus and diaphragm fibres after chronic hypoxia. *Pflugers Arch.* 460 863–873. 10.1007/s00424-010-0866-5 20697736

[B17] Egner I. M. Bruusgaard J. C. Gundersen K. (2016). Satellite cell depletion prevents fiber hypertrophy in skeletal muscle. *Development* 143 2898–2906. 10.1242/dev.134411 27531949

[B18] Eriksson A. Kadi F. Malm C. Thornell L. E. (2005). Skeletal muscle morphology in power-lifters with and without anabolic steroids. *Histochem. Cell Biol.* 124 167–175. 10.1007/s00418-005-0029-5 16059740

[B19] Eriksson A. Lindstrom M. Carlsson L. Thornell L. E. (2006). Hypertrophic muscle fibers with fissures in power-lifters; fiber splitting or defect regeneration? *Histochem. Cell Biol.* 126 409–417. 10.1007/s00418-006-0176-3 16625366

[B20] Fatouros I. G. Kambas A. Katrabasas I. Nikolaidis K. Chatzinikolaou A. Leontsini D. (2005). Strength training and detraining effects on muscular strength, anaerobic power, and mobility of inactive older men are intensity dependent. *Br. J. Sports Med.* 39 776–780. 10.1136/bjsm.2005.019117 16183776 PMC1725040

[B21] Fluck M. Kramer M. Fitze D. P. Kasper S. Franchi M. V. Valdivieso P. (2019). Cellular aspects of muscle specialization demonstrate genotype - phenotype interaction effects in athletes. *Front. Physiol.* 10:526. 10.3389/fphys.2019.00526 31139091 PMC6518954

[B22] Folland J. P. Williams A. G. (2007). The adaptations to strength training : morphological and neurological contributions to increased strength. *Sports Med.* 37 145–168. 10.2165/00007256-200737020-00004 17241104

[B23] Gasier H. G. Fluckey J. D. Previs S. F. Wiggs M. P. Riechman S. E. (2012). Acute resistance exercise augments integrative myofibrillar protein synthesis. *Metabolism* 61 153–156. 10.1016/j.metabol.2011.07.001 21864869

[B24] Gillooly J. F. Hein A. Damiani R. (2015). Nuclear DNA content varies with cell size across human cell types. *Cold Spring Harb. Perspect. Biol.* 7:a019091. 10.1101/cshperspect.a019091 26134319 PMC4484964

[B25] Gokhin D. S. Ward S. R. Bremner S. N. Lieber R. L. (2008). Quantitative analysis of neonatal skeletal muscle functional improvement in the mouse. *J. Exp. Biol.* 211 837–843. 10.1242/jeb.014340 18310108

[B26] Goldspink G. (1964). The combined effects of exercise and reduced food intake on skeletal muscle fibers. *J. Cell. Comp. Physiol.* 63 209–216. 10.1002/jcp.1030630211 14151089

[B27] Goldspink G. (1970). The proliferation of myofibrils during muscle fibre growth. *J. Cell Sci.* 6 593–603. 4911694 10.1242/jcs.6.2.593

[B28] Goldspink G. (2011). Alterations in myofibril size and structure during growth, exercise, and changes in environmental temperature. *Compr. Physiol.* 2 539–554.

[B29] Grgic J. Schoenfeld B. J. (2018). Are the hypertrophic adaptations to high and low-load resistance training muscle fiber type specific? *Front. Physiol.* 9:402. 10.3389/fphys.2018.00402 29720946 PMC5915697

[B30] Haun C. T. Vann C. G. Mobley C. B. Osburn S. C. Mumford P. W. Roberson P. A. (2019a). Pre-training skeletal muscle fiber size and predominant fiber type best predict hypertrophic responses to 6 weeks of resistance training in previously trained young men. *Front. Physiol.* 10:297. 10.3389/fphys.2019.00297 30971942 PMC6445136

[B31] Haun C. T. Vann C. G. Mobley C. B. Roberson P. A. Osburn S. C. Holmes H. M. (2018). Effects of graded whey supplementation during extreme-volume resistance training. *Front. Nutr.* 5:84. 10.3389/fnut.2018.00084 30255024 PMC6141782

[B32] Haun C. T. Vann C. G. Osburn S. C. Mumford P. W. Roberson P. A. Romero M. A. (2019b). Muscle fiber hypertrophy in response to 6 weeks of high-volume resistance training in trained young men is largely attributed to sarcoplasmic hypertrophy. *PLoS One* 14:e0215267. 10.1371/journal.pone.0215267 31166954 PMC6550381

[B33] Haun C. T. Vann C. G. Roberts B. M. Vigotsky A. D. Schoenfeld B. J. Roberts M. D. (2019c). A critical evaluation of the biological construct skeletal muscle hypertrophy: size matters but so does the measurement. *Front. Physiol.* 10:247. 10.3389/fphys.2019.00247 30930796 PMC6423469

[B34] Henderson C. A. Gomez C. G. Novak S. M. Mi-Mi L. Gregorio C. C. (2017). Overview of the muscle cytoskeleton. *Compr. Physiol.* 7 891–944. 10.1002/cphy.c160033 28640448 PMC5890934

[B35] Jorgenson K. W. Hornberger T. A. (2019). The overlooked role of fiber length in mechanical load-induced growth of skeletal muscle. *Exerc. Sport Sci. Rev.* 47 258–259. 10.1249/jes.0000000000000198 31524787 PMC6750012

[B36] Kadi F. Schjerling P. Andersen L. L. Charifi N. Madsen J. L. Christensen L. R. (2004). The effects of heavy resistance training and detraining on satellite cells in human skeletal muscles. *J. Physiol.* 558 1005–1012. 10.1113/jphysiol.2004.065904 15218062 PMC1665027

[B37] Kirby T. J. Patel R. M. Mcclintock T. S. Dupont-Versteegden E. E. Peterson C. A. Mccarthy J. J. (2016). Myonuclear transcription is responsive to mechanical load and DNA content but uncoupled from cell size during hypertrophy. *Mol. Biol. Cell* 27 788–798. 10.1091/mbc.e15-08-0585 26764089 PMC4803305

[B38] Kraemer W. J. Zatsiorsky V. M. (2006). *Science and Practice of Strength Training.* Champaign, IL: Human Kinetics.

[B39] Lee Y. S. Ondrias K. Duhl A. J. Ehrlich B. E. Kim D. H. (1991). Comparison of calcium release from sarcoplasmic reticulum of slow and fast twitch muscles. *J. Membr. Biol.* 122 155–163. 10.1007/bf01872638 1716686

[B40] Lepper C. Partridge T. A. Fan C. M. (2011). An absolute requirement for Pax7-positive satellite cells in acute injury-induced skeletal muscle regeneration. *Development* 138 3639–3646. 10.1242/dev.067595 21828092 PMC3152922

[B41] Luthi J. M. Howald H. Claassen H. Rosler K. Vock P. Hoppeler H. (1986). Structural changes in skeletal muscle tissue with heavy-resistance exercise. *Int. J. Sports Med.* 7 123–127. 10.1055/s-2008-1025748 2942497

[B42] Macdougall J. D. Sale D. G. Elder G. C. Sutton J. R. (1982). Muscle ultrastructural characteristics of elite powerlifters and bodybuilders. *Eur. J. Appl. Physiol. Occup. Physiol.* 48 117–126. 10.1007/bf00421171 7199447

[B43] Mccarthy J. J. Mula J. Miyazaki M. Erfani R. Garrison K. Farooqui A. B. (2011). Effective fiber hypertrophy in satellite cell-depleted skeletal muscle. *Development* 138 3657–3666. 10.1242/dev.068858 21828094 PMC3152923

[B44] Meijer J. P. Jaspers R. T. Rittweger J. Seynnes O. R. Kamandulis S. Brazaitis M. (2015). Single muscle fibre contractile properties differ between body-builders, power athletes and control subjects. *Exp. Physiol.* 100 1331–1341. 10.1113/ep085267 26388513

[B45] Meng H. Janssen P. M. Grange R. W. Yang L. Beggs A. H. Swanson L. C. (2014). Tissue triage and freezing for models of skeletal muscle disease. *J. Vis. Exp.* 89:51586. 10.3791/51586 PMC4215994 25078247

[B46] Metzger J. M. Moss R. L. (1987). Shortening velocity in skinned single muscle fibers. Influence of filament lattice spacing. *Biophys. J.* 52 127–131. 10.1016/s0006-3495(87)83197-1 3607220 PMC1329992

[B47] Mitchell C. J. Churchward-Venne T. A. Cameron-Smith D. Phillips S. M. (2015). What is the relationship between the acute muscle protein synthesis response and changes in muscle mass? *J. Appl. Physiol.* 118 495–497. 10.1152/japplphysiol.00609.2014 25257869

[B48] Mitchell C. J. Churchward-Venne T. A. West D. W. Burd N. A. Breen L. Baker S. K. (2012). Resistance exercise load does not determine training-mediated hypertrophic gains in young men. *J. Appl. Physiol.* 113 71–77. 10.1152/japplphysiol.00307.2012 22518835 PMC3404827

[B49] Mollenhauer H. H. (1993). Artifacts caused by dehydration and epoxy embedding in transmission electron microscopy. *Microsc. Res. Tech.* 26 496–512. 10.1002/jemt.1070260604 8305727

[B50] Moore D. R. Tang J. E. Burd N. A. Rerecich T. Tarnopolsky M. A. Phillips S. M. (2009). Differential stimulation of myofibrillar and sarcoplasmic protein synthesis with protein ingestion at rest and after resistance exercise. *J. Physiol.* 587 897–904. 10.1113/jphysiol.2008.164087 19124543 PMC2669978

[B51] Morpurgo B. (1897). Ueber Activitäts-Hypertrophie der willkürlichen Muskeln. *Arch. Pathol. Anat. Physiol. Klin. Med.* 1S0 522–554.

[B52] Murach K. A. Dungan C. M. Peterson C. A. Mccarthy J. J. (2019). Muscle fiber splitting is a physiological response to extreme loading in animals. *Exerc. Sport Sci. Rev.* 47 108–115. 10.1249/jes.0000000000000181 30640746 PMC6422761

[B53] Murach K. A. Englund D. A. Dupont-Versteegden E. E. Mccarthy J. J. Peterson C. A. (2018). Myonuclear domain flexibility challenges rigid assumptions on satellite cell contribution to skeletal muscle fiber hypertrophy. *Front. Physiol.* 9:635. 10.3389/fphys.2018.00635 29896117 PMC5986879

[B54] Neurohr G. E. Terry R. L. Lengefeld J. Bonney M. Brittingham G. P. Moretto F. (2019). Excessive cell growth causes cytoplasm dilution and contributes to senescence. *Cell* 176:1083. 10.1016/j.cell.2019.01.018 30739799 PMC6386581

[B55] Pansarasa O. Rinaldi C. Parente V. Miotti D. Capodaglio P. Bottinelli R. (2009). Resistance training of long duration modulates force and unloaded shortening velocity of single muscle fibres of young women. *J. Electromyogr. Kinesiol.* 19 e290–e300. 10.1016/j.jelekin.2008.07.007 18801662

[B56] Penman K. A. (1969). Ultrastructural changes in human striated muscle using three methods of training. *Res. Q.* 40 764–772. 10.1080/10671188.1969.10614916 5262107

[B57] Phillips S. M. (2000). Short-term training: when do repeated bouts of resistance exercise become training? *Can J. Appl. Physiol.* 25 185–193. 10.1139/h00-014 10932036

[B58] Reidy P. T. Borack M. S. Markofski M. M. Dickinson J. M. Fry C. S. Deer R. R. (2017). Post-absorptive muscle protein turnover affects resistance training hypertrophy. *Eur. J. Appl. Physiol.* 117 853–866. 10.1007/s00421-017-3566-4 28280974 PMC5389914

[B59] Roberts M. D. Romero M. A. Mobley C. B. Mumford P. W. Roberson P. A. Haun C. T. (2018). Skeletal muscle mitochondrial volume and myozenin-1 protein differences exist between high versus low anabolic responders to resistance training. *PeerJ* 6:e5338. 10.7717/peerj.5338 30065891 PMC6065464

[B60] Roberts M. D. Young K. C. Fox C. D. Vann C. G. Roberson P. A. Osburn S. C. (2020). An optimized procedure for isolation of rodent and human skeletal muscle sarcoplasmic and myofibrillar proteins. *J. Biol. Methods* 7:e127. 10.14440/jbm.2020.307 PMC7081056 32201709

[B61] Roche S. M. Gumucio J. P. Brooks S. V. Mendias C. L. Claflin D. R. (2015). Measurement of maximum isometric force generated by permeabilized skeletal muscle fibers. *J. Vis. Exp.* 100:e52695. 10.3791/52695 PMC4545153 26131687

[B62] Schiaffino S. Hanzlikova V. Pierobon S. (1970). Relations between structure and function in rat skeletal muscle fibers. *J. Cell Biol.* 47 107–119. 10.1083/jcb.47.1.107 5513549 PMC2108409

[B63] Shoepe T. C. Stelzer J. E. Garner D. P. Widrick J. J. (2003). Functional adaptability of muscle fibers to long-term resistance exercise. *Med. Sci. Sports Exerc.* 35 944–951. 10.1249/01.mss.0000069756.17841.9e 12783042

[B64] Soltani M. Vargas-Garcia C. A. Antunes D. Singh A. (2016). Intercellular variability in protein levels from stochastic expression and noisy cell cycle processes. *PLoS Comput. Biol.* 12:e1004972. 10.1371/journal.pcbi.1004972 27536771 PMC4990281

[B65] Stone M. H. Wilson D. R. R. Newton H. (1983). Physiological basis. *Strength Cond. J.* 5 40–64.

[B66] Stromer M. H. (1998). The cytoskeleton in skeletal, cardiac and smooth muscle cells. *Histol. Histopathol.* 13 283–291. 9476658 10.14670/HH-13.283

[B67] Taber C. B. Vigotsky A. Nuckols G. Haun C. T. (2019). Exercise-induced myofibrillar hypertrophy is a contributory cause of gains in muscle strength. *Sports Med.* 49 993–997. 10.1007/s40279-019-01107-8 31016546

[B68] Thys T. M. Blank J. M. Coughlin D. J. Schachat F. (2001). Longitudinal variation in muscle protein expression and contraction kinetics of largemouth bass axial muscle. *J. Exp. Biol.* 204 4249–4257. 11815649 10.1242/jeb.204.24.4249

[B69] Timson B. F. Bowlin B. K. Dudenhoeffer G. A. George J. B. (1985). Fiber number, area, and composition of mouse soleus muscle following enlargement. *J. Appl. Physiol.* 58 619–624. 10.1152/jappl.1985.58.2.619 3980364

[B70] Toth M. J. Miller M. S. Vanburen P. Bedrin N. G. Lewinter M. M. Ades P. A. (2012). Resistance training alters skeletal muscle structure and function in human heart failure: effects at the tissue, cellular and molecular levels. *J. Physiol.* 590 1243–1259. 10.1113/jphysiol.2011.219659 22199163 PMC3381828

[B71] Trappe S. Godard M. Gallagher P. Carroll C. Rowden G. Porter D. (2001). Resistance training improves single muscle fiber contractile function in older women. *Am. J. Physiol. Cell Physiol.* 281 C398–C406. 11443039 10.1152/ajpcell.2001.281.2.C398

[B72] Trappe S. Williamson D. Godard M. Porter D. Rowden G. Costill D. (2000). Effect of resistance training on single muscle fiber contractile function in older men. *J. Appl. Physiol.* 89 143–152. 10.1152/jappl.2000.89.1.143 10904046

[B73] Valle X. Til L. Drobnic F. Turmo A. Montoro J. B. Valero O. (2013). Compression garments to prevent delayed onset muscle soreness in soccer players. *Muscles Ligaments Tendons J.* 3 295–302. 24596693 PMC3940503

[B74] Vann C. G. Osburn S. C. Mumford P. W. Roberson P. A. Fox C. D. Sexton C. L. (2020a). Skeletal muscle protein composition adaptations to 10 weeks of high-load resistance training in previously-trained males. *Front. Physiol.* 11:259. 10.3389/fphys.2020.00259 32292355 PMC7135893

[B75] Vann C. G. Roberson P. A. Osburn S. C. Mumford P. W. Romero M. A. Fox C. D. (2020b). Skeletal muscle myofibrillar protein abundance is higher in resistance-trained men, and aging in the absence of training may have an opposite effect. *Sports* 8:7. 10.3390/sports8010007 31936810 PMC7022975

[B76] Walker D. K. Dickinson J. M. Timmerman K. L. Drummond M. J. Reidy P. T. Fry C. S. (2011). Exercise, amino acids, and aging in the control of human muscle protein synthesis. *Med. Sci. Sports Exerc.* 43 2249–2258. 10.1249/mss.0b013e318223b037 21606874 PMC3289515

[B77] Ward S. R. Eng C. M. Smallwood L. H. Lieber R. L. (2009). Are current measurements of lower extremity muscle architecture accurate? *Clin. Orthop. Relat. Res.* 467 1074–1082. 10.1007/s11999-008-0594-8 18972175 PMC2650051

[B78] Widrick J. J. Stelzer J. E. Shoepe T. C. Garner D. P. (2002). Functional properties of human muscle fibers after short-term resistance exercise training. *Am. J. Physiol. Regul. Integr. Comp. Physiol.* 283 R408–R416. 12121854 10.1152/ajpregu.00120.2002

[B79] Wilkens J. L. Cavey M. J. Shovkivska I. Zhang M. L. Ter Keurs H. E. (2008). Elasticity, unexpected contractility and the identification of actin and myosin in lobster arteries. *J. Exp. Biol.* 211 766–772. 10.1242/jeb.007658 18281339

[B80] Wilkinson D. J. Franchi M. V. Brook M. S. Narici M. V. Williams J. P. Mitchell W. K. (2014). A validation of the application of D(2)O stable isotope tracer techniques for monitoring day-to-day changes in muscle protein subfraction synthesis in humans. *Am. J. Physiol. Endocrinol. Metab.* 306 E571–E579. 24381002 10.1152/ajpendo.00650.2013 PMC3948971

[B81] Yamada A. Yoshio M. Nakayama H. (1997). Bi-directional movement of actin filaments along long bipolar tracks of oriented rabbit skeletal muscle myosin molecules. *FEBS Lett.* 409 380–384. 10.1016/s0014-5793(97)00558-9 9224694

[B82] Yu J. G. Liu J. X. Carlsson L. Thornell L. E. Stal P. S. (2013). Re-evaluation of sarcolemma injury and muscle swelling in human skeletal muscles after eccentric exercise. *PLoS One* 8:e62056. 10.1371/journal.pone.0062056 23614012 PMC3626686

